# Treatment of a Complex Case of Catatonia and Conversion Features With Electroconvulsive Therapy in a 14-Year-Old Male

**DOI:** 10.31486/toj.19.0026

**Published:** 2020

**Authors:** Cody Roi, Luke Verret, Bradley Peet, Erich J. Conrad

**Affiliations:** ^1^ Department of Psychiatry, Louisiana State University Health Sciences Center, New Orleans, LA; ^2^ Louisiana State University School of Medicine, New Orleans, LA

**Keywords:** *Catatonia*, *conversion disorder*, *electroconvulsive therapy*, *mental disorders*

## Abstract

**Background:** Pediatric catatonia is a rare and poorly understood phenomenon. The majority of reported cases have a psychiatric etiology. Because of the heterogeneous presentation and treatment issues unique to the pediatric population, identification and management can be challenging. Additionally, few definitive guidelines or practice parameters are available for pediatric patients. The first-line treatment for catatonia is pharmacologic, and when treatment fails or is inadequate, electroconvulsive therapy (ECT) has been shown to be safe and effective.

**Case Report:** A previously healthy, 14-year-old male presented with acute onset of catatonia that resolved at 4 weeks after a short course of ECT with adjunctive lorazepam and risperidone. An interesting feature of this case was the resolution of autonomic symptoms and the emergence of conversion features. The resolution of the catatonia (negativism, mutism, and withdrawal) made it possible for the team to identify a thought disorder and initiate appropriate pharmacologic treatment for the precipitating etiology.

**Conclusion:** ECT was a safe and effective treatment for the resolution of catatonia symptoms in this patient. Conversion and catatonia features may exist on a continuum.

## INTRODUCTION

Catatonia is a complex neuropsychiatric syndrome characterized by a marked psychomotor disturbance that may involve either increased or decreased motor activity.^1^ Pediatric catatonia is associated with many disorders, including psychiatric, neurologic, and general medical conditions (Table 1),^2,3^ but is associated predominately with mood disorders and schizophrenia.^4^

**Table 1.** Psychiatric and Nonpsychiatric Etiologies of Catatonia^2,3^

Psychiatric Etiologies	Nonpsychiatric Medical Condition Etiologies
Thought disorders Schizophrenia Schizoaffective disorder Brief psychotic disorder Schizophreniform disorder	Autoimmune Autoimmune encephalitis NMDA-receptor encephalitis Autoimmune thyroiditis Opsoclonus-myoclonus syndrome
Mood disorders Bipolar disorder Major depressive disorder	Neurologic Temporal lobe epilepsy Status epilepticus Lesion of thalamus or parietal or frontal lobe Bilateral globus pallidus disease Head trauma Kleine-Levin syndrome
Neurodevelopmental disorders Autism spectrum disorder	Metabolic Diabetic ketoacidosis Homocystinuria Hypercalcemia Hepatic encephalopathy Hyponatremia
Medication induced Neuroleptics	Genetic Prader-Willi syndrome
	Medication induced Corticosteroids, immunosuppressants

NMDA, N-methyl-D-aspartate.

The *Diagnostic and Statistical Manual of Mental Disorders, Fifth Edition* (*DSM-5*) specifies 12 core symptoms of catatonia: stupor, catalepsy, waxy flexibility, mutism, negativism, posturing, mannerisms, stereotypy, agitation, grimacing, echolalia, and echopraxia.^1^ These symptoms are categorized as retarded, excited, or malignant. The retarded form frequently presents with symptoms such as posturing, rigidity, mutism, and repetitive movements. The excited form is characterized by restless movements, talkativeness, and agitation. Fink and Taylor described the malignant form, which is the most severe form and the most likely to be fatal, to be associated with autonomic instability, including hypertension, tachycardia, tachypnea, and hyperthermia.^5^

Theories of etiology and treatment recommendations have primarily been informed by the adult literature. Although research on catatonia in the pediatric population is limited, some differences vs catatonia in adults have been identified: boys are more affected than girls (2:1 ratio), schizophrenia spectrum disorders are the most frequent cause (compared to mood disorders in adults), and the prevalence of traumatic events precipitating the onset is potentially increased.^6^

In addition to the challenges of recognizing and treating the catatonic state, clinicians must also elucidate the prodromal or primary etiology. Psychiatric diagnoses appear to represent the majority of cases in the pediatric population,^3^ and, regardless of the precipitant etiology, treatment recommendations are similar. However, once medical causes have been ruled out, making an accurate diagnosis of underlying psychiatric conditions in catatonic patients continues to be a challenge because of (1) the heterogeneous and more liberal list of diagnostic criteria for catatonia that now includes 3 of 12 potential symptoms in the *DSM-5* compared to 3 of 5 in the *DSM-4*, (2) the broad differential for catatonia, (3) diagnostic overshadowing for primary precipitating psychiatric diagnosis (eg, schizophrenia vs autism, depression vs anxiety), and (4) the potentially lengthy course of catatonia, with variable mental status examination findings throughout. In the pediatric population, an additional confounding factor is that children have less developmental history than adults for comparison of findings.

Lorazepam is recommended as first-line treatment in catatonia,^7^ although successful treatment with other benzodiazepines has been reported.^8-10^ If the patient shows no response to benzodiazepines in 48 to 72 hours, electroconvulsive therapy (ECT) is recommended.^11^

Catatonia is thought to be attributable to dysregulation of γ-aminobutyric acid (GABA)–releasing neurons and dysregulation of dopaminergic and glutaminergic systems, and both benzodiazepines and ECT appear to exert their effects through GABA systems.^12,13^ ECT has been used in children and adolescents primarily for refractory major depression. ECT is generally considered safe, but an increase in the frequency of prolonged seizures is possible.^14^ ECT has been shown to be highly effective for the treatment of catatonia in adults and adolescents and to lack serious side effects.^15-18^

## CASE REPORT

A 14-year-old Asian American male with no psychiatric or medical history presented with progressive deterioration from baseline function during the prior 5 weeks, including withdrawal from social activities, apathy, periods of mutism, wandering out of the house, and perseveration on religious themes not congruent with his baseline beliefs or behavior. On presentation to the emergency department, he was mute and unable to walk without assistance. He was initially treated with a course of lorazepam (Ativan) for suspected catatonia and appeared to improve. However, upon his initial transfer to the psychiatric hospital, he became significantly more catatonic with mutism, negativism, and stupor. His initial medical workup was negative and consisted of routine laboratory tests, head imaging (computed tomography and magnetic resonance imaging), lumbar puncture with cerebrospinal fluid cytology, gram stain and culture, encephalitis panel, enterovirus polymerase chain reaction, anti-N-methyl-D-aspartate receptor panel, human immunodeficiency virus, rapid plasma reagin, hepatitis panel, Lyme serology, antinuclear antibodies, urine toxicology, urinalysis and culture, thyroid panel, acetaminophen level, salicylate level, C-reactive protein, vitamin B12, ammonia, partial thromboplastin time, prothrombin time and international normalized ratio, and magnesium. Video electroencephalography showed no evidence of nonconvulsive status epilepticus and showed normal awake, drowsy, and sleep structure. Because of the patient's catatonia, a nasogastric tube and intravenous (IV) line were started to maintain nutrition and hydration as he had stopped taking food and medications orally.

The patient was given lorazepam 2 mg IV and had a rapid response, including opening his eyes and following commands. However, he quickly returned to a catatonic state, including mutism, negativism, and stupor, as well as eyelid fasciculations, slowness to constrict pupils, and the formation of copious amounts of frothy saliva that he refused to expel from his mouth, thought to be associated with a psychotic thought process and best described as psychogenic sialorrhea. Idiopathic paroxysmal sialorrhea has been described in the literature but primarily in relation to medical causes.^19^

Lorazepam was administered to the patient for the next 10 days and titrated up to 2 mg every 4 hours. This treatment provided some relief initially. The patient was observed conversing briefly with his family, but within 24 hours, he returned to stupor and subsequently responded very little to the lorazepam. On day 11, amantadine was added to his regimen and titrated up to 100 mg twice daily with no improvement (Figure).

**Figure.**
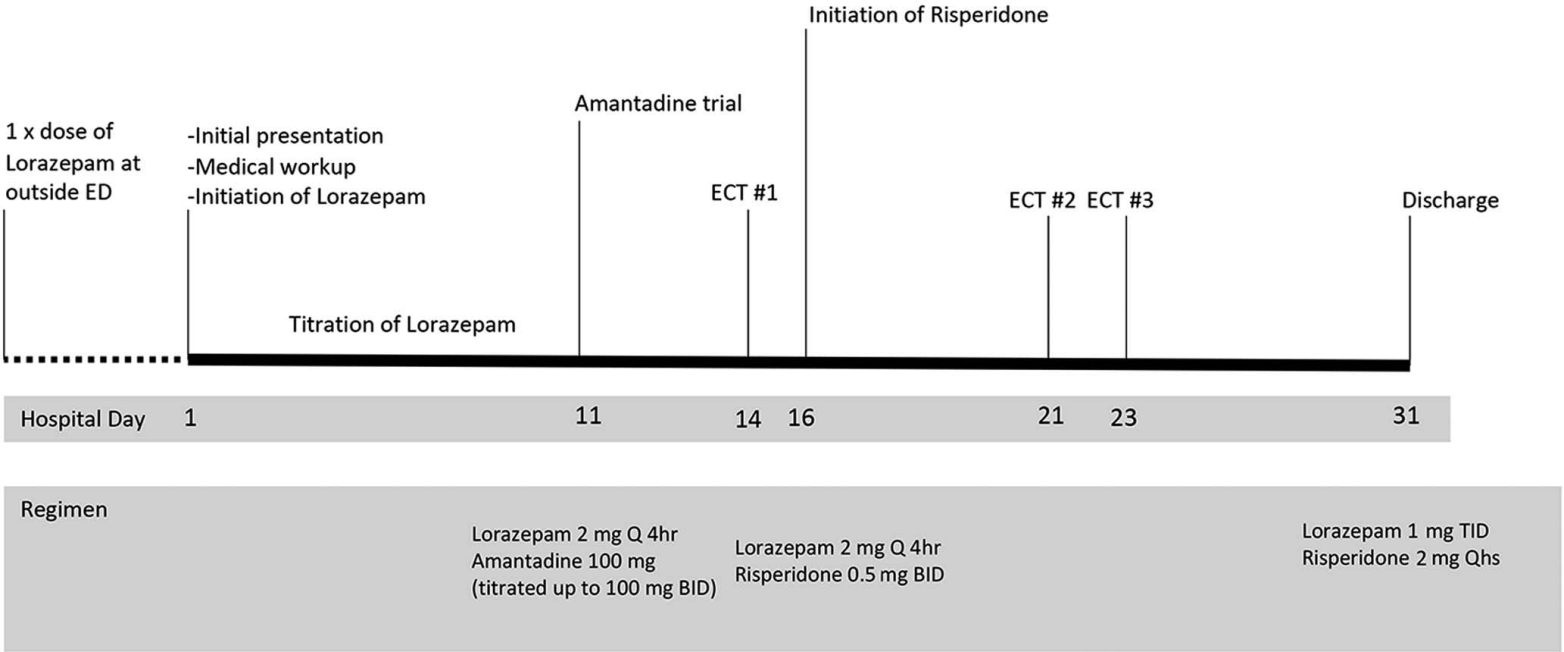
**Timeline of treatments.** BID, twice daily; ECT, electroconvulsive therapy; ED, emergency department; Q, every; Qhs, nightly; TID, three times daily.

As the patient's nutritional status deteriorated, ECT was arranged to be performed at a nearby hospital, and he received his first treatment on day 14. Prior to the first treatment, the patient was evaluated by 2 child and adolescent psychiatrists: an ECT psychiatrist and a psychiatrist with expertise in religions to ensure that the odd statements the patient made about religious beliefs during lucid moments were not a culturally appropriate variant. Multiple family meetings took place to obtain informed consent.

Utilizing a Thymatron System IV (Somatics, LLC), the patient underwent ECT with bifrontal electrode placement at 5% energy without a seizure, with propofol 80 mg and succinylcholine 50 mg. A second stimulus at 10% induced a satisfactory seizure with good quality for 71 seconds. He tolerated the treatment well. Forty-eight hours after his first ECT treatment, the patient was noted to be near his baseline behavior, per his parents, and he was maintaining proper hydration and nutrition. Lorazepam was continued, and amantadine was stopped.

The patient was transferred back to the psychiatric hospital for further assessment of his primary psychiatric condition. He returned to a catatonic state with mutism and echopraxia. Risperidone 0.5 mg twice daily was started. On hospital day 21, the patient received a second ECT treatment at 10% energy for 35 seconds, and 2 days later, he received a third ECT treatment at 15% energy with resultant improvement of catatonia. On day 31, the patient was discharged home on lorazepam 1 mg 3 times daily and risperidone 2 mg at night. For the first 2 weeks at home, the patient had continued periods of paranoid and disorganized thinking, as well as transient periods of mutism.

During the subsequent 6 weeks, his risperidone dose was titrated up to 3 mg daily in divided doses, and lorazepam was tapered down to 1 mg twice daily. At 8-week follow-up, the patient remained free of catatonic episodes, had reintegrated into a home school program, and began to resume his previous recreational activities. At 10 weeks postdischarge, the patient's caregivers reported a near recovery to his premorbid level of function, and he was maintained on risperidone monotherapy 2 mg nightly.

## DISCUSSION

In this case, the decision to use ECT was difficult to make. Information on options for treatment and evidence has been limited until fairly recently^6^ and is not widely disseminated among practicing clinicians. The patient's incomplete response to lorazepam and worsening nutritional status warranted additional intervention. The team was surprised at his dramatic response to the first treatment when he returned to near his premorbid baseline but was disappointed that the single treatment response was not sustained. However, further treatments resulted in the ultimate relief of the catatonia. The delay between the first ECT treatment and the second and third ECT treatments was because the patient had variable and fluctuating examination findings after the first ECT treatment. In addition to his catatonic states, which initially included mutism, stupor, negativism, autonomic changes, eyelid fasciculation, and periods of enuresis, the patient also had periods during which he was mute but without autonomic fluctuations and with apparent volitional attempts to resist physical examination (Table 2).

**Table 2.** Symptom Evolution and Comparison at Various Phases of Treatment

		Symptoms Subsequent to ECT Treatment 1 (Two Fluctuating States)		
Symptoms Prior to Catatonia	Symptoms Prior to ECT Treatment 1	State 1	State 2	Symptoms Subsequent to ECT Treatment 3
Paranoia Social withdrawal Odd behaviors Culturally incongruent beliefs	Negativism Mutism Stupor Psychogenic sialorrhea Heart rate and blood pressure variability Eyelid fasciculations Slowness to constrict pupils Echopraxia Enuresis	Negativism Mutism Stupor Eyelid fasciculations	Mutism Psychogenic sialorrhea Volitional withdrawal Paranoia	Paranoia

ECT, electroconvulsive therapy.

The persistent fluctuation and dramatic disparity between the 2 apparently distinct states—the catatonia with autonomic changes and the nonresponsive volitional periods—led the team to consider 3 possibilities: (1) the patient had 2 distinct states of fluctuation between catatonia and a conversion process, (2) the patient's presentation was a primary conversion disorder in which the neurologic manifestation was the catatonia, or (3) the catatonia and conversion symptoms existed as a single etiologic process on a continuum.

The most dramatic symptoms to respond to the ECT appeared to be the autonomic findings (Table 2). The implications of understanding this fluctuation are important and may help better inform the psychoneurologic phenomenon of catatonia and conversion and inform the indication for ECT in pediatric populations. This diagnostic difficulty has also been noted in adults.^20,21^ However, Leong and colleagues have noted the utility of ECT for conversion as well.^22^

## CONCLUSION

ECT was an effective and safe treatment that resolved the patient's symptoms of pediatric catatonia. Clinicians should consider using ECT in similar cases.

## Notes

This article meets the Accreditation Council for Graduate Medical Education and the American Board of Medical Specialties Maintenance of Certification competencies for Patient Care and Medical Knowledge.
